# Collaborative learning about e-health for mental health professionals and service users in a structured anonymous online short course: pilot study

**DOI:** 10.1186/1472-6920-12-37

**Published:** 2012-05-31

**Authors:** Emily J Ashurst, Ray B Jones, Graham R Williamson, Tobit Emmens, Jon Perry

**Affiliations:** 1 Faculty of Health, Education and Society, University of Plymouth, Drake Circus, Plymouth, Devon, PL4 8AA, UK; 2 Research and Development, Devon Partnership NHS Trust, Wonford House Hospital, Dryden Road, Exeter, EX2 5AF, UK

## Abstract

**Background:**

Professionals are interested in using e-health but implementation of new methods is slow. Barriers to implementation include the need for training and limited awareness or experience. Research may not always convince mental health professionals (MHPs). Adding the 'voice' of mental health service users (MHSUs) in collaborative learning may help. Involving MHSUs in face-face education can be difficult. We had previously been unable to engage MHPs in online discussion with MHSUs. Here we assessed the feasibility of short online courses involving MHSUs and MHPs.

**Methods:**

We ran three e-health courses, comprising live interactive webcast, week’s access to a discussion forum, and final live interactive webcast. We recruited MHPs via posters, newsletters, and telephone from a local NHS trust, and online via mailing lists and personal contacts from NHS trusts and higher education. We recruited MHSUs via a previous project and an independent user involvement service. Participants were presented with research evidence about e-health and asked to discuss topics using professional and lived experience. Feasibility was assessed through recruitment and attrition, participation, and researcher workloads. Outcomes of self-esteem and general self-efficacy (MHSUs), and Internet self-efficacy and confidence (MHPs) were piloted.

**Results:**

Online recruiting was effective. We lost 15/41 from registration to follow-up but only 5/31 that participated in the course failed to complete follow-up. Nineteen MHPs and 12 MHSUs took part and engaged with each other in online discussion. Feedback was positive; three-quarters of MHPs indicated future plans to use the Internet for practice, and 80% of MHSUs felt the course should be continued. Running three courses for 31 participants took between 200 to 250 hours. Before and after outcome measures were completed by 26/31 that participated. MHP Internet self-efficacy and general Internet confidence, MHSU self-esteem and general self-efficacy, all seemed reliable and seemed to show some increase.

**Conclusions:**

Collaborative learning between MHSUs and MHPs in a structured online anonymous environment over a one-week course is feasible, may be more practical and less costly than face-face methods, and is worthy of further study.

## Background

E-health, the use of the Internet and mobile devices, has benefited mental health service users (MHSUs) in a variety of studies and new methods continue to be developed [1]. Findings have highlighted many positive effects of e-health methods such as computerised cognitive behavioural therapy for reducing depression and anxiety [2], online forums for social support [3], and web-based interventions for improved health behaviours [4]. E-health methods can be as effective as traditional face-face therapies [5], be cost effective [6] and have greater reach to rural communities [7]. MHSUs increasingly turn to the Internet for information, advice, support and to share personal experiences [8], and expect health services to offer Internet provisions [9]. In particular, we are likely to see mobile ‘apps’ being effectively used by younger cohorts [10,11].

Professionals are interested in using e-health but implementation of new methods is slow [12,13]. Frequently cited barriers to implementing e-health include a need for training [14] and limited awareness or experience [15]. Mental health professionals (MHPs) may overestimate the negative effects of e-health on patients [16]. Patients have often been more positive about telecare and e-health than providers [17], and for example, surveys show that an overwhelming numbers of clients wish to contact their clinicians by email but few clinicians wish to provide that [18]. Professionals need more evidence of the benefits e-health can offer their patients before adoption of methods [19].

Rogers’ ideas on the diffusion of innovation [20] are well known. Potential adopters of new technologies, such as e-health, must first learn about the innovation, perhaps see some local demonstration of it, and be persuaded to try it out before deciding to adopt or reject. As a first stage, therefore, we need to do more in continuing professional education for health professionals both to introduce research evidence but also to give them the opportunity to hear of personal experience.

MHSUs may have more or different experience in the use of the Internet for their healthcare and this expertise could help inform MHPs. Involvement in MHPs’ education may also be therapeutic for MHSUs [21]. Patient involvement in the education of healthcare professionals has yielded positive outcomes both for learners in terms of satisfaction and learning experiences, and for patients in terms of self-esteem and empowerment for sharing their experiences to benefit future health service delivery [22]. We have not found examples of user involvement in online learning, yet potentially given that both MHSUs and MHPs can be drawn from a national ‘pool’, involving no travel for either, and in an environment in which power differentials between them are reduced, ‘online’ should be easier and less costly to organise.

We had tried to involve MHPs in discussion forums with young people who self-harm but found that MHPs were uncomfortable with the format and failed to participate [23]. We hypothesised that two contributing factors were: (i) the lack of focus on a particular topic and (ii) the expectation of participation over several months. Here, therefore, we have explored the feasibility of a short online ‘course’ for MHPs to learn about various e-health methods via anonymous online discussions with MHSUs. We were concerned whether we would be able to recruit both MHPs and MHSUs, and whether they would be willing to participate fully in the discussions. We aimed to test the feasibility of this approach and to assess whether self-esteem for MHSUs and Internet self-efficacy for MHPs were appropriate measures.

## Methods

### Ethics

The study was approved by the South West 2 Research Ethics Committee of the National Health Service (NHS).

### Recruitment

We wanted participation from UK-based MHPs interested in learning more about Internet methods for practice and MHSUs who were confident users of the Internet for their mental health. Our target was up to twenty MHPs and eight MHSUs for each course. Recruitment was online. Visitors to the project website could watch a video about the project, or download information sheets. Those wishing to participate completed an online registration form, gave consent by entering their email address, and provided an anonymous username for use during the course.

To raise awareness of the project website among MHPs we used three methods: (i) With a local mental health NHS Trust, we used posters, Trust newsletter, personal emails and follow up telephone calls to adult mental health team managers. (ii) Emails were sent to all 200 staff members of the relevant faculty of our own university, with the request to further distribute to relevant contacts, (iii) emails were sent to two email lists (jiscmail consumer-health-informatics list and Patient Information Forum) and (iii) emails to individuals identified from the websites of UK wide NHS mental health trusts.

We raised awareness among MHSUs using two methods: (i) We contacted participants from a previous project, who had expressed an interest in collaborating in further research [23]. (ii) We emailed the development worker from a local independent mental health involvement service. She circulated the advert via online newsletter to approximately 200 MHSUs and 190 related service workers.

### Intervention

We ran three courses, each comprising a live interactive webcast, a week’s access to a discussion forum, and a final live interactive webcast. The course comprised discussions on 12 topics on e-health methods for mental health services: (i) computerised cognitive behavioural therapy (CCBT) for depression, (ii) discussion forums, (iii) lifestyle change intervention websites, (iv) webcast group therapy, (v) videophone, (vi) email, (vii) computer-patient interviews, (viii) Map of Medicine, (ix) patient access to their online medical records, (x) barriers to greater use of Internet in mental health, (xi) groups who would benefit from Internet, and (xii) implementation and requirements for supporting Internet uses. Five topics were introduced in the first webcast and the rest via the forum. In most cases we presented a brief overview and some examples of relevant research publications but the main focus was on participant discussion. The final webcast was used to review the forum discussions and for the last three topics.

All participants were co-learners, identified only by usernames. The organisers and online environment gave no indication as to whether participants were MHPs or MHSUs. Participants however may have chosen to reveal this in their own comments in discussion.

Registrants were emailed course outline, passwords, and a step-by-step course guide document. Documents included sections for troubleshooting technical issues, instructions for testing computer video and sound capabilities, and a guide to e-health terminology. Personalised reminders were sent a day before the course.

The discussion forum was created using phpBB, open source bulletin board software [24], and the live webcasts were delivered using a combination of hardware and software described elsewhere [25]. The first webcast slides and initial set of topics were posted to the discussion forum.

Participants logged into the live webcast (Additional file 1: Appendix 1) via the project website 15 min before the hour long live presentation at 6 pm. An MHP and the principal investigator (RBJ) presented the webcast. The presentation involved descriptions of some successful uses of e-health for mental health with intervals for typed discussions between participants. We used ‘break out rooms’ if needed, to keep the number of participants in any group below eight.

The researcher (EJA) assisted with any technical queries from participants during webcasts by private message or email. Technical issues included login trouble, inability to view webcast video, Internet connection problems, and frozen screen. These were resolved within webcast sessions.

At the end of the one-hour live interactive webcast, a personalised email was sent to remind participants of discussion forum access details. MHPs were requested to post a minimum of 5 posts to the discussion forum to gain the course completion certificate.

The discussion forum was accessed via the project website and was accessible at any time until the following live interactive webcast. Webcast topics and subsequent chat transcripts were posted to the discussion forum for continued discussion (Additional file 1: Appendix 2), and evidence about e-health interventions from published literature was added to forum topics for further reading and discussion. The day before the final webcast, participants were sent a personalised email reminder. On the seventh and final day of the course, there was a second live interactive webcast presented with summaries of the week’s topic discussions with opportunity for further discussion.

### Incentives

For taking part in the course MHPs were emailed a Certificate of Course Completion for their continuing professional development records and MHSUs were emailed a £10 e-voucher for participating.

### Email follow-up

Directly after, participants were emailed asking for their views on the course (content, convenience, timing, duration, course materials), for any changes they would recommend, and recruitment. MHPs were asked if they would recommend the course to a colleague.

### Before and after questionnaire measures

Online questionnaires were developed using LimeSurvey [26] an open source survey application. Participants were emailed a link to the baseline questionnaire before the course, and two weeks after the course a link to the follow-up questionnaire.

The baseline questionnaire for MHPs measured Internet self-efficacy in a 4-item scale (Additional file 1: Appendix 3) developed from a previous study [27] that employed elements of an Internet self-efficacy scale by Barnoy [28]. As a comparison between the scale and self-rated Internet self-efficacy, participants were also asked to rate (on a scale of 1–10) their general confidence in using the Internet for mental health practice. Additional questions pertained to e-health attitudes and awareness and elicited open text responses. The follow-up questionnaire repeated these questions with additional questions about future plans for using the Internet in practice and views on evidence to support e-health benefits to patients.

The baseline questionnaire for MHSUs measured trait self-esteem, state self-esteem and self-efficacy. Trait self-esteem was measured using Rosenberg’s 10-item Self Esteem Scale [29]. This scale is a frequently used measure of self-esteem, however a criticism is that it only measures trait self-esteem, so we also employed Heatherton’s 20-item state self-esteem scale [30]. Self-efficacy was measured using Schwarzer & Jerusalem’s 10-item General Self Efficacy scale [31]. The follow up questionnaire repeated these questions with additional questions of the course utility. The general self-efficacy questions were not included in the follow-up, but instead were sent by email and included baseline responses for additional qualitative feedback to gauge if any differences observed ‘made sense’ to them and if the course had influenced their self-esteem.

### Workloads

Time spent on different activities was estimated retrospectively from analysis of project documentation and emails.

## Results

### Recruitment

We recruited 23 MHPs (of which 19 participated) and 18 MHSUs (of which 12 participated). Online methods (direct to known contacts, circulation & online searches for contact addresses, and online newsletter) were effective for MHP recruitment, recruiting 19 participants with about 15 hours work. Non-online methods (paper newsletter, posters, leaflets, and telephone calls) were not effective with no recruits for a similar effort (Additional file 1: Appendix 4). The two online methods of contacting MHSUs were both effective (Additional file 1: Appendix 5) requiring less than one hour in total from the research team.

### Participant characteristics

Most participants were female, 13/19 MHPs and 10/12 MHSUs. The mean age of MHPs (42) and MHSUs (41) was similar. MHPs were from various parts of the UK. Their occupations included trainees (in clinical psychology, cognitive behavioural therapy, and psychological wellbeing), nurses, occupational therapists, lecturers and researchers (Table 1). Fifteen participants were from NHS Trusts, three from UK Universities and one from the US. MHSUs had used mental health services for depression, self-harm and mood and personality disorders. Almost all MHSUs had prior experience with discussion forums and social networks, most had experience with instant chat messaging but fewer with Internet telephony, video or photo posting and virtual world experience (Table 1).

**Table 1:** Characteristics of 19 mental health professionals and 12 mental health service users who participated

**MHP (n = 19)**		**MHSU (n = 12)**	
Characteristic	n	Characteristic	n
Sex		Sex	
Male	6	Male	2
Female	13	Female	10
Age		Age	
25–29	2	16–20	3
30–39	4	21–30	2
40–49	11	31–40	1
50–59	1	41–50	1
60+	1	51–60	2
		61–70	3
Occupation		Condition	
Trainee	5	Depression	4
Nurse	3	Depression with other condition	3
Occupational Therapist	2	Self-harm with other condition	2
Lecturer	2	Mood disorder	1
Researcher	2	Personality disorder	1
Mental Health Social worker	1	Non specified	1
Clinical Psychologist	1		
Dietician (adult mental health)	1	Internet experience	
Senior Mental Health Practitioner	1	Discussion forum	11
Team Manager	1	Social networking	11
		Instant chat	8
		Internet telephony	5
		Post video or photo	3
		Virtual world	2

### Participation by course

Two MHPs and six MHSUs took part in the first, six MHPs and five MHSUs took part in the second and 11 MHPs and five MHSUs took part in the third course. Four MHSUs participated in more than one course. Both MHPs and MHSUs posted a similar number of times: averages of 11 by MHPs and 13 by MHSUs during webcasts, and 7 by MHPs and 5 by MHSUs on the forum.

### Attrition and participation in the courses

From initial registration through to completion of follow-up questionnaire we lost 15/41 (37%) but most of this loss was from registration to participation (10/41) (Table 2). Only 5/31 that participated failed to complete the follow-up questionnaire. Of those who participated in the course, involvement was comparable between MHPs and MHSUs (Additional file 1: Appendix 8), with 95% and 92% respectively taking part in the first webcast (mean of 14 comments from both) and discussion forum (mean of 7 posts from MHPs and 5 from MHSUs), and 84% and 67% respectively participating in the final webcast (mean of 8 comments from MHPs and 14 from MHSUs).

**Table 2:** MHP and MHSU attrition rates from recruitment to follow up questionnaire completion

**Participant**	**Recruited**	**Completed baseline**	**Participated in course**	**Gave feedback**	**Completed follow-up**
MHP	23 (100%)	22 (96%)	19 (83%)	18 (78%)	16 (70%)
MHSU	18 (100%)	17 (94%)	12 (67%)	10 (56%)	10 (56%)
All	41 (100%)	39 (95%)	31 (76%)	28 (68%)	26 (63%)

### Outcome measures for MHPS

Internet self-efficacy showed some improvement in this small sample (Table 3), from a mean of 3.2 to 3.5 at follow-up (*p* = .14), and general Internet confidence showed significant improvement, from 6.4 to 7.8 at follow-up with a medium effect size (*F*(1,15) = 6.51, *p* = .02, η^2^ = .30). There was a significant positive correlation between the four-item and the one-item scale at baseline (*r* = .670, p = .005) but not at follow up. The reliability coefficient of the 4-item internet self-efficacy scale was acceptable (α = .778) at baseline but much lower at follow-up (α = .310). There did not appear to be any ceiling effects in the use of these variables (Additional file 1: Appendix 6).

**Table 3:** Mean MHP Internet self-efficacy and general Internet confidence scores at baseline and follow up (n = 16)

	**Baseline**	**Follow up**
	***M (SD)***	***M (SD)***
Internet self-efficacy (4-item scale; range 1-5)	3.2 (.81)	3.5 (3.75)
Items within scale:		
Confidence searching webpages to find evidence to help with a consultation with a patient	3.9 (0.96)	4.2 (0.66)
Confidence about potentially running a live interactive webcast or chat room for a group of patients (but were anonymous to each other)	2.8 (1.05)	2.9 (1.06)
Confidence about potentially using Internet video telephony (e.g. Skype) for remote consultations with individual patients	3.3 (1.15)	3.4 (0.73)
Confidence about potentially running a discussion forum for a group of patients	3.0 (1.03)	3.6 (0.81)
General Internet confidence (1-item scale, range 1-10)	6.4 (2.36)	7.8 (0.78)

Higher scores indicate higher self-efficacy.

The increase in Internet self-efficacy scores was reflected in answers to open questions giving face validity to the measures. From comments, awareness of Internet based mental health therapies appeared to increase for 6/16 MHPs based on greater numbers of referenced sources and self-reported awareness increase (e.g. reporting ‘not aware of any’ at baseline and reporting ‘*yes Mind Gym and other CBT programmes. Online assessment and screening tools*’ at follow-up).

### Outcome measures for MHSUs

Amongst MHSUs trait self-esteem increased significantly from baseline to follow-up with a medium effect size (*F*(1,9) = 7.11), *p* = .026, η^2^ = .44), as did general self-efficacy with a large effect size (*F*(1,5) = 9.42, *p* = .028, η^2^ = .65) (Table 4). Trait self-esteem, state self-esteem, and self-efficacy were all correlated both at baseline and follow-up. The reliability coefficients were all high both at baseline and follow-up for trait self-esteem scale (.93 and .93), state self-esteem scale (.97 and .97), and general self-efficacy scale (.96 and .95). There did not appear to be any ceiling or floor effects (Additional file 1: Appendix 7).

**Table 4:** Mean MHSU scores at baseline and at follow-up for trait self-esteem (score range of 1–4); state self-esteem (score range of 1–5); general self-efficacy (score range of 1–4), higher scores indicate higher self-esteem & self-efficacy

	**Baseline**	**Follow up**	***n***
Trait self-esteem	2.2 (0.59)	2.3 (0.67)	10
Sate self-esteem	2.8 (1.04)	2.8 (0.89)	10
General self-efficacy	2.6 (0.74)	2.8 (0.77)	6

### Other measures

At follow-up, 12/16 MHPs indicated plans for future use of the Internet with patients. Proposed uses included signposting to self-help websites, use of discussion forums, email & Skype communications, and continued development of own software. A quarter (4/16) did not indicate future plans due to workplace restrictions and one MHP indicated further evidence was needed to make an informed choice. Three-quarters (12/16) thought that there was good evidence that Internet use in mental health practice benefitted patients. Most (8/10) MHSUs at follow up felt the course should continue to be offered and 7/10 thought the course was successful.

### Email follow-up

Email responses from 18/19 MHPs and 10/12 MHSUs following the course suggested that both MHPs and MHSUs considered the course helpful, interesting and enjoyable, and commented that hearing other’s views was a great opportunity. Some MHPs found the course helpful for exploring new methods and helpful for future use in practice and one MHSU found the course helpful for reflecting on their treatment methods. Some MHPs thought fewer topics and longer webcast sessions might be better. Most MHPs would recommend the course to colleagues and five reportedly had done so. Most MHPs suggested online methods to recruit other professionals to future courses. Both MHPs and MHSUs said that an initial attraction to the course was an interest in e-health methods and/or the course delivery method. MHPs also mentioned an opportunity for skills development. Some MHSUs wanted to help make a change to health care and have the opportunity to ‘give back’ for mental health care received.

### Illustrative workloads

In addition to the 30 hours raising awareness for these courses we spent 16 hours recruiting and joining participants (registering each participant on discussion forum and sending course instructions, login details, and links to questionnaires), and 22 hours in providing support and reminders (responses to general course comments and queries), during the three courses. We spent 19 hours concluding the courses with follow up questionnaires, awarding course completion certificates and e-vouchers and offering a summary of findings. In addition, undocumented time was spent preparing and delivering the webcasts, moderating and contributing to the discussion forum, searching for additional literature in response to forum discussions, and dealing with recordings of webcasts and the transfer of transcripts. We estimate the total time spent recruiting and running these three courses for 31 participants as between 200–250 hours.

## Discussion

### Overall need for, and impact of, this approach

There is a large variation across the UK in adoption of e-health, both in mental and other health services. For example, online cognitive behavioural therapy site for depression using Living Life to the Full, shows marked variation by postcode area probably dependent on enthusiasm from health professions in some regions to recommend it [32]. UK General Practitioners vary in the availability and functionality of their websites. For example, in 2011, while practices such as Haughton Thornley Medical Centres in Cheshire [33] provide information, repeat prescribing, appointment booking, online advice, and access to their own medical record, others have no website at all.

Online methods may be cheaper, more acceptable to some, and more ‘environmentally friendly’ than face-face methods such as focus groups. Many MHSUs would be intimidated in a face-face meeting with professionals but feel comfortable contributing anonymously online. Travel time and cost for all participants can be less online and the discussion is automatically transcribed. For the asynchronous parts (discussion forum) participants can contribute when convenient to them. However, we had not found reports of collaborative online learning for MHPs with MHSU.

This pilot study demonstrated the feasibility of recruiting both professionals and service users to take part and collaborate in a one-week anonymous online course. The course was well received by participants based on feedback comments, and attrition from participation to follow-up was low. The outcome measures found promising results for improving e-health knowledge and the confidence of professionals, and apparent improvement in self-esteem of service users, suggesting further study with a fully powered sample size is worthwhile. We have shown elsewhere by a discourse analysis that MHSUs and MHPs ‘engaged’ in the online discussion [34].

Most professionals in this pilot study said they now had plans to implement e-health measures into their practice. Those who did not, reported external limitations such as trust policy or wanted to further explore e-health ideas first. Although this was only a small scale pilot study, those intentions and the increase in Internet self-efficacy suggest that online courses like this, based on anonymous discussion of e-health topics with service users, may be effective.

MHSUs too reported satisfaction with their involvement and the increment, in this small sample, in MHSU trait self-esteem and self-efficacy scores suggests further study of this approach may be worthwhile, although problems with measures of self-esteem are discussed further below.

### Recruitment and operational considerations

Much effort was put into recruiting professionals(30 hours for 23 recruits) but half, the time spent on posters and leaflets, seemed unproductive. We had expected greater numbers of registrants and considered possible limitations were time constraints and other commitments, which were the main reason given by MHPs who were least active in the course. The most effective MHP recruitment method was by direct email to known contacts, many of whom reported further circulating to their known contacts. Participant numbers increased as we ran courses suggesting a ‘snowball’ effect as advert awareness spread. Online searches for email contacts appeared reasonably effective but was quite labour intensive and some form of online advertising may be more cost-effective [35,36].

We tried running the webcast in the early evening hoping that participants would access it using their home computers, as we knew that some NHS local networks struggle or prohibit access to such live video streams [25]. Nevertheless, although this was an optimal time for some, for others the timing of the live webcasts was difficult. On the other hand our experience in other studies (Unpublished results from New Dynamics of Ageing, ‘Grey and Pleasant Land’ project [37]) suggests that having an ‘event’ is important to gain participation and that completely asynchronous contacts have lower priority. One possible alternative may be to use a ‘semi-synchronous’ approach where a recorded (rather than live) video stream is available and discussion is within certain hours. That approach may lose much of the ‘social presence’ [38] of live webcasting but such compromises may be worth trying.

Although we were able to refine both interface and guidelines for use of webcasting and the forum we still had a substantial number of technical queries, suggesting that more work is needed.

### Suitability of outcome measures for further study

The single question on Internet self-efficacy for MHPs, when asked subsequent to the four-item scale and open questions about their use of e-health, seemed reasonably appropriate. The correspondence with answers to open questions and the MHPs’ plans to implement e-health suggest the question has some validity. A variety of Internet self-efficacy scales have been used by others, for example, in 2000 Eastin and LaRose reported an eight-item scale with reliability coefficient of .93 [39]. In recent years, the eHEALS measure of e-health literacy [40] has been increasingly used, although normally for patients [41] rather than professionals. Like most self-efficacy scores it can be criticised on the grounds that respondents may have poor insight into their ability [42]. For this reason, and based upon our experience of using self-efficacy scales previously [27], we think that lead-in questions to any ‘self-confidence’ question are needed to consistently ‘ground’ people’s views in reality. Although our four item scale did not show good reliability at follow-up, it helps to make respondents give a more realistic answer to the one-item self-efficacy question. Whether to use this approach or the eHEALS approach needs further review before use in a definitive trial.

The measures of self-esteem for MHSUs, at first sight, seemed appropriate. For example, Rush and Barker found improved self-esteem amongst MHSUs [43]. Role reversal, the consequent shift in power dynamics, the confidence derived from involvement in academic activity and positive feedback may be the main causes of a rise in self-esteem. Trait self-esteem, state self-esteem, and general self-efficacy scales were all reliable, comparable with, or better than, previous studies [30,44,45]. On the other hand, while trait self-esteem (which ‘logically’ should not change) seemed to show improvement, state self-esteem scores on average remained the same. The ‘illogical’ use of change in trait self-esteem by many authors was criticised by Heatherton and Polivy, and was the motivation for their development of a measure of state self-esteem [30]. Their measure has been widely used by others (cited over 500 times in Web of Science November 2011). However, perhaps other approaches are needed. Harter and Whitesell argued that self-esteem and other constructs are not, in and of themselves, trait-like or state-like in nature. Rather, certain individuals display trait-like behaviour, whereas others demonstrate change in self-esteem or self-worth across relatively long periods of time, on a short-term basis, and across situations [46].

### Limitations

MHP participants were self-selected and mostly represented those with a prior interest in e-health methods so we cannot know how an online course such as this would be accepted by other MHPs.

Recruiting MHSUs was very easy in this study as we were able to recruit some from a previous study. That experience may not generalise to other locations or times. However, other MHSUs were recruited via a ‘Be Involved’ organisation; such organisations are found in most parts of the UK.

## Conclusions

This pilot study suggests that MHSUs and MHPs are prepared to work together in a structured online environment over a one-week course. Online recruitment was successful and ‘traditional’ methods were not. The method appears feasible, participants were satisfied with the experience and would recommend the course, so it seems worthy of further study. The outcome measures of self-esteem amongst MHSUs and Internet self-efficacy amongst MHPs appear appropriate.

## Competing interests

The authors declare that they have no competing interests.

## Authors' contributions

RBJ had the idea for the study and was the principal investigator. GRW and TE were co-investigators and co-grant holders. EJA was the appointed researcher and was responsible for the day to day running of the project supervised by RBJ, GRW, TE. JP helped with some of the interaction with users and contributed to the literature. EJA and RBJ carried out the analysis and wrote the paper which was edited and approved by all authors.

## Pre-publication history

The pre-publication history for this paper can be accessed here:

http://www.biomedcentral.com/1472-6920/12/37/prepub

## Supplementary Material

Additional file 1 **Appendix 1.** Live webcast: Screenshot example of live webcast presentation window and live chat between participants. Additional file 1: Appendix 2: Discussion forum: Screenshot example of MHSU and MHP discussion forum posts. Additional file 1: Appendix 3: MHP Internet self-efficacy scale: 4-item questionnaire to measure MHP Internet self-efficacy on a 5-point scale of ‘not at all confident’ to ‘totally confident’. Additional file 1: Appendix 4: MHP Recruitment methods: Table of methods used for recruiting MHP participants, including workload time estimates and outcome. Additional file 1: Appendix 5: MHSU Recruitment methods: Table of methods used for recruiting MHSU participants, including workload time estimates and outcome. Additional file 1: Appendix 6: MHP ceiling effects: Distribution of MHP scores for Internet self-efficacy and general Internet confidence in practice at baseline and at follow up. Additional file 1: Appendix 7: Relationship between MHP scales and plans for subsequent change. Additional file 1: Appendix 8: MHSU ceiling effects: Distribution of MHSU scores for Trait and State self-esteem and general self-efficacy at baseline and follow up. Click here for file
