# Supplementary material for: Collaborative learning about e-health for mental health professionals and service users in a structured anonymous online short course: pilot study
Source: BMC Med Educ. 2012 May 31;12:37. doi: 10.1186/1472-6920-12-37 (PMC3676147; doi:10.1186/1472-6920-12-37)
Supplement: Additional file 1 — Appendix 1. Live webcast: Screenshot example of live webcast presentation window and live chat between participants. Additional file 1: Appendix 2: Discussion forum: Screenshot example of MHSU and MHP discussion forum posts. Additional file 1: Appendix 3: MHP Internet self-efficacy scale: 4-item questionnaire to measure MHP Internet self-efficacy on a 5-point scale of ‘not at all confident’ to ‘totally confident’. Additional file 1: Appendix 4: MHP Recruitment methods: Table of methods used for recruiting MHP participants, including workload time estimates and outcome. Additional file 1: Appendix 5: MHSU Recruitment methods: Table of methods used for recruiting MHSU participants, including workload time estimates and outcome. Additional file 1: Appendix 6: MHP ceiling effects: Distribution of MHP scores for Internet self-efficacy and general Internet confidence in practice at baseline and at follow up. Additional file 1: Appendix 7: Relationship between MHP scales and plans for subsequent change. Additional file 1: Appendix 8: MHSU ceiling effects: Distribution of MHSU scores for Trait and State self-esteem and general self-efficacy at baseline and follow up. [file 1472-6920-12-37-S1.doc]

# Collaborative learning about e-health for mental health professionals and service users in a structured anonymous online short course: pilot study

**Appendix**

### Appendix 1

### Live webcast: Screenshot example of live webcast presentation window and live chat between participants


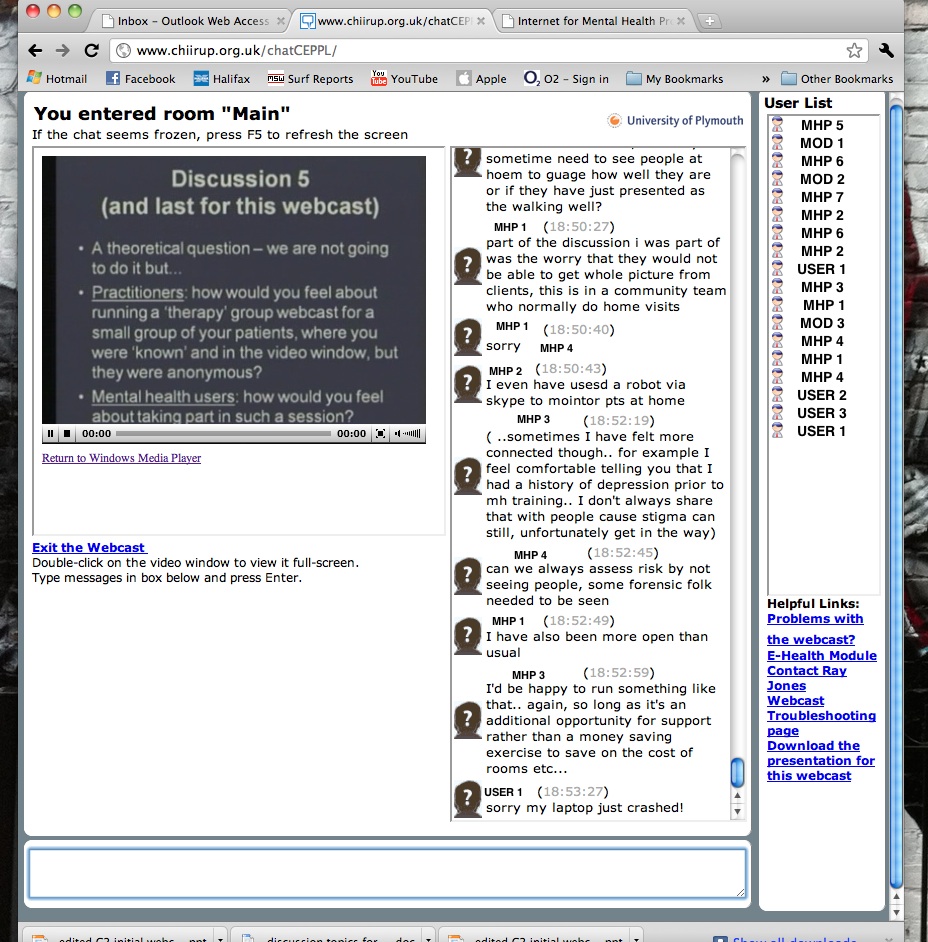


### Appendix 2

### Discussion forum: Screenshot example of MHSU and MHP discussion forum posts


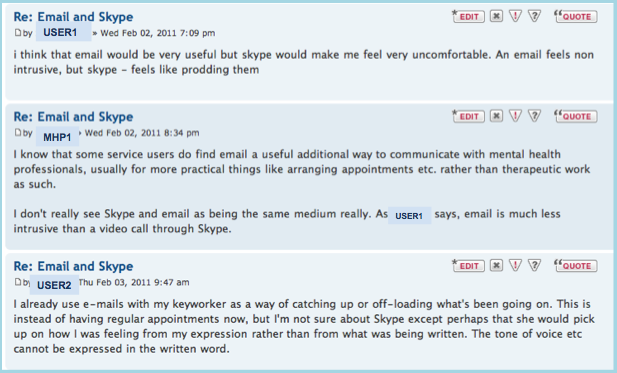


### Appendix 3

### MHP Internet self-efficacy scale: 4-item questionnaire to measure MHP Internet self-efficacy on a 5-point scale of ‘not at all confident’ to ‘totally confident’.

| How confident are you in searching webpages to find evidence to help with a consultation with a patient? |
| --- |
| How confident would you feel about running a live interactive webcast or chat room for a group of your patients (but who were anonymous to each other)? |
| How confident would you feel about using Internet video telephony (eg Skype) for remote consultations with individual patients? |
| How confident would you feel about running a discussion forum for a group of patients? |

### Appendix 4

### MHP Recruitment methods: Table of methods used for recruiting MHP participants, including workload time estimates and outcome

| **Method** | **Total Time**  **(mins)** | **Recruited** | **Participated** |
| --- | --- | --- | --- |
| **1. Email** |  |  |  |
| Direct to known contacts | 120 | 4 | 4 |
| Via known contacts | 15 | 6 | 6 |
| Online search for contacts | 670 | 8 | 4 |
| **2. Online news** |  |  |  |
| Via Trust Communications Manager | 15 | 1 | 1 |
| **3. Paper news** |  |  |  |
| Via Trust Communications Manager | 10 | 0 | 0 |
| **4. A4 poster sent by mail** |  |  |  |
| + follow up phone calls and emails | 480 | 0 | 0 |
| **5. Leaflet distribution** |  |  |  |
| within PU Faculty | 120 | 0 | 0 |
| **6. Poster presentation** |  |  |  |
| within PU Faculty | 240 | 0 | 0 |
| **7. Unknown:** | - | 4 | 4 |
| **TOTAL** | 1670 | 23 | 19 |
|  |  |  |  |

### Appendix 5

### MHSU Recruitment methods: Table of methods used for recruiting MHSU participants, including workload time estimates and outcome

| **Method** | **Total time (minutes)** | **Recruited** | **Participated** |
| --- | --- | --- | --- |
| **Email**  Via previous project | 10 | 4 | 4 |
| Via service user organisation | 15 | 3 | 3 |
| **Unknown** | - | 11 | 5 |

### Appendix 6

MHP ceiling effects: Distribution of MHP scores for Internet self-efficacy and general Internet confidence in practice at baseline and at follow up

| Range of possible scores | Top 25% | Middle 50% | Bottom 25% | 19 completed baseline or 16 who also completed follow-up |
| --- | --- | --- | --- | --- |
| Baseline 4-item Internet self-efficacy | | | | |
| 4 – 20 | 1 | 17 | 1 | 19 |
|  | 1 | 14 | 1 | 16 |
| Follow up 4-item Internet self-efficacy | | | | |
| 4 – 20 | 1 | 15 | 0 | 16 |
| Baseline 1-item Internet self-efficacy | | | | |
| 1 – 10 | 3 | 15 | 1 | 19 |
|  | 3 | 12 | 1 | 16 |
| Follow up 1-item Internet self-efficacy | | | | |
| 1 – 10 | 2 | 14 | 0 | 16 |

**Appendix 7**

Relationship between MHP scales and plans for subsequent change

|  | **4-item scale** | | **1-item scale** | |
| --- | --- | --- | --- | --- |
|  | **No Plan** | **Plan** | **No Plan** | **Plan** |
| **Baseline** | 2.9 | 3.3 | 5.3 | 6.8 |
| **Follow up** | 3.3 | 3.6 | 7.0 | 8.0 |
| **N** | 4 | 4 | 12 | 12 |

### Appendix 8

### MHSU ceiling effects: Distribution of MHSU scores for Trait and State self-esteem and general self-efficacy at baseline and follow up

| Range of possible scores | Top 25% | Middle 50% | Bottom 25% | 12 completed baseline or 10 who also completed follow-up |
| --- | --- | --- | --- | --- |
| Baseline trait self-esteem | | | | |
| 10 – 40 | 0 | 9 | 3 | 12 |
|  | 0 | 7 | 3 | 10 |
| Follow up trait self-esteem | | | | |
| 10 – 40 | 1 | 7 | 2 | 10 |
| Baseline state self-esteem | | | | |
| 20 – 100 | 2 | 8 | 2 | 12 |
|  | 2 | 6 | 2 | 10 |
| Follow up state self-esteem | | | | |
| 20 – 100 | 1 | 7 | 2 | 10 |
| Baseline general self-efficacy | | | | |
| 10 – 40 | 1 | 10 | 1 | 12 |
|  | 1 | 8 | 1 | 10 |
| Follow up general self-efficacy | | | | |
| 10 – 40 | 1 | 4 | 1 | 6 |
